# Nutritional support during the hospital stay is cost-effective for preventing adverse outcomes in patients with cancer

**DOI:** 10.3389/fonc.2022.916073

**Published:** 2022-08-09

**Authors:** Philipp Schuetz, Suela Sulo, Stefan Walzer, Sebastian Krenberger, Cory Brunton

**Affiliations:** ^1^ Medical University Department, Kantonsspital Aarau, Aarau, Switzerland; ^2^ Medical Faculty, University of Basel, Basel, Switzerland; ^3^ Abbott Nutrition, Global Health Economics & Outcomes Research, Chicago, IL, United States; ^4^ MArS Market Access & Pricing Strategy GmbH, Weil am Rhein, Germany; ^5^ State University Baden-Wuerttemberg, Loerrach, Germany; ^6^ Social Work & Health Care, University of Applied Sciences Ravensburg-Weingarten, Weingarten, Germany

**Keywords:** cancer, oncology, inpatient, malnutrition, nutritional treatment, cost-effectiveness

## Abstract

**Objective:**

Among patients with cancer, malnutrition remains common and is a key challenge in oncology practice today. A prior study from our group revealed that malnourished cancer inpatients who got nutritional treatment (intervention group) had lower mortality and improved functional and quality of life outcomes compared to inpatients without nutritional support (control group). Our present analysis aimed to determine whether the improved patient recovery by nutritional support was paralleled by cost-effectiveness of this nutritional care.

**Methods:**

We analyzed hospital costs and health outcomes in patients with cancer, using a Markov simulation model with daily cycles to analyze the economic impact of nutritional support in malnourished inpatients with malignancies. We compared results for a nutritional intervention group and a control group across a 30-day timeframe. Five health states were designated (malnourished but stable, complications, intensive care unit (ICU) admission, discharge, death). Costs for the different health states were based on publicly available data for the Swiss medical system. Total patient cost categories included in-hospital nutrition, days spent in the normal ward, days in the ICU, and medical complications.

**Results:**

Total per-patient costs for in-hospital supportive nutrition was Swiss francs (CHF) 129. Across a 30-day post-admission interval, our model determined average overall costs of care of CHF 46,420 per-patient in the intervention group versus CHF 43,711 in the control group—a difference of CHF 2,709 per patient. Modeled results showed a cost of CHF 1,788 to prevent one major complication, CHF 4,464 to prevent one day in the ICU, and CHF 3,345 to prevent one death. Recovery benefits of nutritional care were thus paralleled by cost-effectiveness of this care.

**Conclusion:**

In-hospital nutritional support for oncology patients at nutritional risk is a low-cost intervention that has both clinical and financial benefits.

## Introduction

Malnutrition is common among patients with cancer today, and this condition remains a key challenge in oncology practice. Too often these conditions go unrecognized and untreated, in turn leading to harmful consequences for patients. Malnutrition develops in at least 30% of patients with malignancies, and its presence is associated with higher mortality, impaired functional status, and longer hospital stays (1–3). Older people with cancer are particularly vulnerable to malnutrition due to their higher risk of cancer and to side-effects of its treatments, compounded by aging-related metabolic changes (2–4). Up to half of all patients with advanced cancer experience cachexia, a severe form of malnutrition characterized by fatigue and loss of weight and muscle mass (5).

Prior studies have shown benefits to identifying patients with malnutrition or its risk at the time of hospital admission, and to providing nutritional support during the hospital stay. In a large, prospective, noncommercial, multicenter, randomized controlled trial conducted in eight hospitals in Switzerland (*Effect of early nutritional support on Frailty, Functional Outcomes, and Recovery of malnourished medical inpatients Trial*, EFFORT), Schuetz et al. examined outcomes of providing nutritional support for such patients (6). In this trial, hospitalized medical patients were randomly assigned to a group that received protocol-guided individualized nutritional support (intervention group) or to one that received standard hospital food (control group); most patients in the intervention group met calorie and protein goals, and they had increased likelihood of 30-day survival. In a secondary analysis of EFFORT trial, Bargetzi et al. focused on a subgroup of oncology patients (N=506 patients with various types of cancer) (7). Results revealed that patients with cancer who received interventional nutritional treatment had lower mortality risk and had improved functional and quality of life outcomes compared to the control group (7). Specifically, within the 30-day timeframe, 36 of 255 intervention-group patients (14.1%) died compared to 50 of 251 control group patients (19.9%), yielding an adjusted odds ratio of 0.57 (95% CI 0.35-0.94; P = 0.027) for mortality.

Despite recognized recovery benefits with nutrition, studies of cost-effectiveness for nutritional care in patients with cancer are scarce (8, 9). The objective of our present analysis was to determine whether the recovery benefits of nutritional support were paralleled by cost-effectiveness of this nutritional care. For our current cost-effectiveness analysis, we examined hospital costs and health outcomes in patients with cancer, using modeling to compare results for the nutritional intervention and control groups, as we have done previously for poorly nourished medical inpatients (10) and for patients with chronic heart failure (11). We used clinical data of patients with cancer included in the EFFORT trial – the largest randomized trial today outside critical care that compared clinical outcomes among patients receiving individualized nutritional support with usual hospital care (6).

## Methods

### Study design

This study was a secondary economic analysis of 506 oncology patients who were part of the EFFORT trial. The trial was registered at ClinicalTrials.gov (https://clinicaltrials.gov/ct2/show/NCT02517476). Of the participating trial patients at risk of malnutrition (Nutritional Risk Screening [NRS], 2002 edition ≥ 3 points), 255 patients had a cancer diagnosis and were randomized to the intervention group (individualized nutritional support to reach energy, protein, and micronutrient goals), while 251 were randomized to the control group (receiving standard hospital food).

Individualized nutritional support included screening patients for malnutrition risk on admission; dietitian-conducted nutritional assessment for patients identified to be at risk for malnutrition; individualized nutritional care plans developed by a dietitian; and implementation of the care plan with monitoring of health outcomes during hospitalization and follow-up post-discharge (6).

The composite primary endpoint of EFFORT trial was defined as any adverse clinical outcome, including all-cause mortality, admission to the intensive care unit (ICU) from the medical ward, non-elective hospital readmission after discharge, and major complications (such as nosocomial infection, respiratory failure, major cardiovascular events, acute renal failure, gastrointestinal events, or a decline in functional status of 10% or more from admission to day 30 as measured by Barthel Index).

### Simulation model

We developed a Markov simulation model with daily cycles to analyze the economic impact of nutritional support in malnourished inpatients with cancer diagnosis/malignancies; the model reflected the perspective of Swiss health insurers. A modeling timeframe of one month (30 days) with five designated health states (malnourished but stable, complications, ICU admission, discharge, death) was based on findings in a recent systematic review and meta-analysis report (12). In the present analysis, we assumed that all patients began in a stable health state—hospitalization with all types of cancer and with evidence of malnutrition risk on admission. The average patient in the model was 69 years or older and at nutritional risk per NRS-2002.

During hospitalization, patients could develop complications, such as infection with multi-resistant pathogens. Worsening symptoms and complications might require transfer to the ICU. Other modeled states included discharge from the hospital and readmission for a non-elective reason. It was assumed that all patients are being released from hospital within the time frame of 30 days with only stable malnutrition patients being released from hospital. It was assumed that those patients would not die within the time of release until day 30. Afterwards those patients had the same probability to die as all other surviving patients. After stable malnutrition patients had been released from hospital, it was assumed that there would be no readmission to the hospital until day 30.

Notably, patients have different costs for care and risk for death in each state. Transition probabilities between health states were based on outcome results for patients with cancer in our full EFFORT trial calculated from day 30 relative risk. The rates per study arm were calculated for each health state and then transferred into daily probabilities. Mean values and standard deviations were calculated for each health state. These were used to estimate the parameters of the beta distribution, which was the assumed distribution for the probabilistic analysis (10).

### Cost determinations

Costs for the different health states were assumed as follows: (i) costs for nutritional support were based on the publication by Schuetz et al., 2020 (10), assuming a standard deviation of 20% of the input values for inpatients and outpatients; (ii) costs for 20% of post-discharge patients to continue nutritional supplements were based on cost data from the largest Swiss online pharmacy (13); (iii) costs for a heterogeneous distribution of adverse events were estimated on the basis of the Swiss Disease-related Group (DRG) (14); (iv) costs for a complex treatment in the case of colonization or infection with multi-resistant pathogens were based on the Swiss DRG costs (14); (v) costs for ICU admission were based on the Swiss DRG costs for complex treatment in ICU (14); (vi) and no costs for death. The scenario assumes hospitals already offer nutritional support for patients (reimbursed as part of the respective Swiss DRG). Hence, only the CHF 5 per day for the nutrition cost per patient was modelled.

Costs to prevent major complications, days in ICU and deaths were calculated with the difference in costs between the intervention and non-intervention group, which was then examined in terms of the difference in life days gained with and without nutritional support.

## Results

Recovery benefits of nutritional care were paralleled by cost-effectiveness of this care (**Table 1**). Across a 30-day post-admission interval, overall costs of care averaged Swiss francs (CHF) 46,420 per-patient in the intervention group versus CHF 43,711 in the control group—a difference of CHF 2,709 per patient. Total costs included cost of in-hospital nutrition (CHF 129), costs for days patients spent in the normal ward and in the ICU, and excess costs due to medical complications. The higher cost for patients who received nutritional support was largely due to more days in normal wards, which may be explained by the lower mortality in these patients.

**Table 1:** Cost-effectiveness of nutritional support vs. no nutritional support in the EFFORT trial oncology subgroup over 30-days. CHF, Swiss franc.

Cost item	Nutritional support	No nutritional support
Nutrition	CHF 129	–
Day in normal ward	CHF 43,593	CHF 40,495
Day in ICU	CHF 843	CHF 1,328
Major complication	CHF 1,853	CHF 1,888
Total	CHF 46,420	CHF 43,711

ICU-Intensive care unit

When we calculated costs to prevent serious adverse outcomes within the 30-day timeframe, we found low costs for important outcome improvements (**Table 2**). Specifically, the cost to prevent one major complication was CHF 1,788, to prevent one day in the ICU was CHF 4,464, and to prevent one death CHF 3,345.

**Table 2:** Cost to prevent adverse outcomes within a 30-day timeframe. CHF, Swiss franc.

Adverse outcome within	Cost
One major complication	CHF 1,788
One day in ICU	CHF 4,464
One death	CHF 3,345

## Discussion

Our modeling results suggest that in-hospital nutritional support for oncology patients at nutritional risk is a low-cost intervention that has both clinical and financial benefits. Total per-patient costs for in-hospital supportive nutrition was CHF 129. Across a 30-day post-admission interval, our model determined average overall costs of care of CHF 46,420 per-patient in the intervention group versus CHF 43,711 in the control group—a difference of CHF 2,709 per patient. Modeled results showed a cost of CHF 1,788 to prevent one major complication, CHF 4,464 to prevent one day in the ICU, and CHF 3,345 to prevent one death. Recovery benefits of nutritional care were thus paralleled by cost-effectiveness of this care. Taken together, these findings strongly support the inclusion of nutritional care when treating patients hospitalized with cancer and malnourished or at risk of malnutrition.

### The impact of malnutrition in cancer

While malnutrition develops in about one third of patients with malignancies, up to 70% may experience malnutrition at some time over the course of their disease (15). Muscaritoli et al. showed that impaired nutritional status was evident as early as the patient’s first visit to a medical oncology center; in fact, 51% had nutritional impairment, 43% were considered at risk of malnutrition, and 9% were overtly malnourished (16). The likelihood of malnutrition depends on patient age, type of cancer, and stage of cancer (3, 17, 18). Patients who are older age (3), have head and neck, lung, or gastrointestinal tumors (2), and have advanced tumor stages (19) are most likely to experience malnutrition and its adverse consequences.

Poor nutritional status and weight loss can lead to poor outcomes for patients with cancer, including decreased quality of life, decreased functional status, increased complication rates, and treatment disruptions (2, 3, 20–22). Further, it has been estimated that up to 20% of patients with cancer deaths can be attributed to malnutrition rather than to the cancer disease (23). By contrast, nutritional interventions—nutritional counseling, oral nutritional supplements, and enteral or parenteral nutrition—can reverse these adverse effects and improve functionality, decrease complications, and increase survival (1–3, 24)

In general, hospital malnutrition has been established as a critical, prevalent, and costly problem (25). Among oncology patients, the financial burden for healthcare systems to provide added care is also high. For example, in a study of the economic impact of malnutrition in patients who underwent surgery for colorectal cancer, Melchior et al. found that malnourished patients had a mean LOS in hospital 3.41 days significantly longer than well-nourished patients, with the cost of hospital stay increased by about €3360 (26).

Nutritional interventions have clear benefits in terms of both health and cost savings. A recent study of patients with head and neck cancer found that dietary counseling and ONS may improve quality of cancer care at no additional costs, although further research on the cost-effectiveness of nutritional supplementation was recommended (9). On the other hand, in a cost-effectiveness analysis of dietary supplementation in cancer survivors, Shaver et al. found that hospitalization rates for nutritional supplement users and non-users were 12% and 21%, respectively; the cost of hospitalization was $4030. Supplementation was associated with an additional 0.48 quality-associated life years (QALYs, 10.26 vs. 9.78) at the incremental cost of $2094 ($236,933 vs. $234,839) over the remaining lifetime of survivors (average 13 years) (9). Schuetz et al. recently applied cost estimates (27) to the outcome results from their earlier systematic review and meta-analysis of hospitalized patients (27 trials, n=6803 patients) (12). Results showed that costs of care within the model timeframe of 6 months averaged US$63,227 per patient in the nutrition intervention group versus US$66,045 in the control group, which corresponded to per-patient cost savings of US$2818. Such savings were mostly due to reduced infection rate and shorter lengths of stay; costs to prevent a hospital-acquired infection and a non-elective readmission were US$820 and US$733, respectively. The incremental cost per life-day gained was -US$1,149 with 2.53 additional days (27).

Further, despite high rates of malnutrition in hospitalized patients, a recent report noted that malnutrition diagnoses were not coded in at least one-third of cases, thus leading to lost reimbursements for hospital care (28).

### Study limitations

As for all modeling analyses, our model had limitations as previously reported (10, 11). Of note, the calculation of costs and cost savings was informed from the perspective of the Swiss hospitals included in the original EFFORT trial (29), which may not be representative of other hospitals worldwide. Additionally, our modelled cost-savings calculations reflect reductions in major complications, ICU admission and death with other clinical and treatment related outcomes of interest (e.g., treatment interruptions, rehospitalizations, etc.) not included in the analysis. Due to the relatively small numbers of patients, no subgroup analyses based on stage of cancer, cancer type, and/or treatment modality were performed. Finally, the outcomes of interest were modelled for a 30-day period which is a relatively short period of time considering the nature of cancer with significant variations in healthcare resource use and therefore costs. Future economic analysis can benefit from employing longer-term modelling periods.

## Conclusion

Taken together, our findings showed that in-hospital nutritional support for oncology patients at nutritional risk is a low-cost intervention that has strong clinical and financial benefits. Compared to other more invasive procedures, nutritional support can help protect patients from adverse events that require cost-intensive care.

## Data availability statement

The data that support the findings of this study are not openly available due to the sensitive nature of the data and are available from the corresponding author upon reasonable request.

## Funding

The initial EFFORT trial was funded by the Swiss National Science Foundation (SNSF) (PP00P3_150531) and the Research Council of the Kantonsspital Aarau (1410.000.058 and 1410.000.044). Abbott provided a grant (HA34) to cover expenses associated with the economic analysis.

## Acknowledgments

We thank Cecilia Hofmann, PhD of C. Hofmann & Associates for manuscript review and editing.

## Conflict of interest

SW and SK were employed by MArS Market Access & Pricing Strategy GmbH. SS and CB were employed by Abbott Laboratories.

The remaining authors declare that the research was conducted in the absence of any commercial or financial relationships that could be construed as a potential conflict of interest.

The authors declare that this study received funding from Abbott Nutrition. The funder had the following involvement with the study: writing of this article.

## Publisher’s note

All claims expressed in this article are solely those of the authors and do not necessarily represent those of their affiliated organizations, or those of the publisher, the editors and the reviewers. Any product that may be evaluated in this article, or claim that may be made by its manufacturer, is not guaranteed or endorsed by the publisher.
